# Intussusception caused by cecal duplication cyst mimicking appendicitis: A case report

**DOI:** 10.1016/j.radcr.2025.07.026

**Published:** 2025-08-06

**Authors:** Zineb Yammouri, Zineb Essolaymany, Hajar Ouazzani Chahdi, Ismail Chaouche, Amal Akammar, Nizar El Bouardi, Badreddine Alami, Moulay Youssef Alaoui Lamrani, Mustapha Maaroufi, Meryem Boubbou

**Affiliations:** a Department of Mother and Child Radiology, CHU Hassan II Fez, Sidi Mohammed Ben Abdellah University, Fez, Morocco; b Department of Adult Radiology, CHU Hassan II Fez, Sidi Mohammed Ben Abdellah University, Fez, Morocco

**Keywords:** Caecal duplication, Intestinal duplication, Intestinal intussusception

## Abstract

The cecum is an uncommon location for intestinal duplication cysts. They are usually identified within the first 2 years of a child's life. Symptoms can vary, but often include nausea, stomach pain, bloating, a noticeable lump, and rectal bleeding. Because the symptoms are usually vague, these cysts can be challenging to diagnose and typically require a combination of clinical suspicion and imaging techniques. Ultrasound, computed tomography (CT), and magnetic resonance imaging (MRI) are key diagnostic tools for medical imaging. Although rare, these cysts must be identified early to prevent bowel obstruction, perforation, or volvulus. Few cases of cecal duplication have been reported. In this report, we discuss a case involving a 4-year-old girl who presented with frequent episodes of intermittent abdominal pain, but no signs of blockage or vomiting. Upon examination, tenderness was noted in her lower right abdomen, which prompted further diagnostic evaluation. Imaging confirmed that she had an intestinal intussusception due to a cecal duplication cyst. This case underscores the significance of considering cecal duplication cysts when diagnosing persistent abdominal pain in children, as prompt detection and surgery can avert serious complications.

## Background

Cecal duplication cysts are an uncommon birth defect of the digestive system, accounting for approximately 0.4% of all gastrointestinal duplications [1,2]. These cysts are notable for their close connection with the nearby intestine, sharing a common layer of smooth muscle, inner lining, and the same blood supply. The clinical presentation of cecal duplication cysts varies depending on their size, type, and anatomical location. Patients may present with symptoms including an abdominal mass, intussusception, gastrointestinal hemorrhage, or abdominal pain mimicking acute appendicitis, as well as chronic or acute intestinal obstruction [[3], [4], [5]]. In most instances, symptoms appear within the first 2 years of a child’s life, making timely radiological tests crucial for proper diagnosis and treatment [6]. Imaging modalities such as ultrasonography, computed tomography, and magnetic resonance imaging are essential for detecting and characterizing these lesions. In this article, we present a case study of an infrequent occurrence of intussusception over a duplication cyst in the cecum. The management of this case involved a thorough clinical examination, followed by additional diagnostic tests. An initial abdominal ultrasound enabled the diagnosis.

## Case presentation

**Fig. 1:**
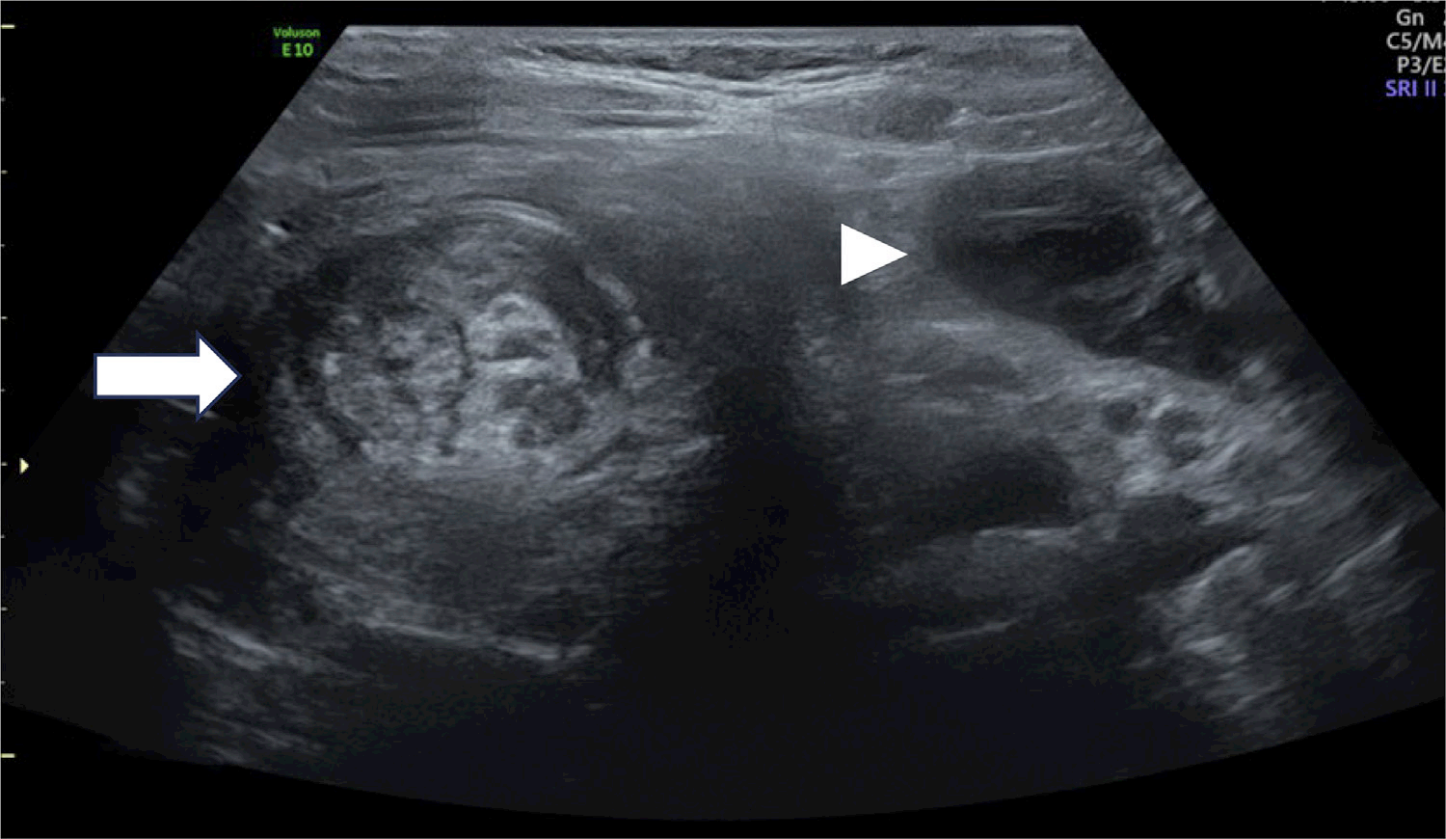
Transverse ultrasound image of the right iliac fossa showing the classic “*target*” appearance of ileocolic intussusception (white arrow), characterized by concentric hypoechoic and hyperechoic bowel wall layers. Adjacent to the intussusception, a well-defined cystic structure (white arrowhead) is visualized, suggestive of a cecal duplication cyst.

**Fig. 2:**
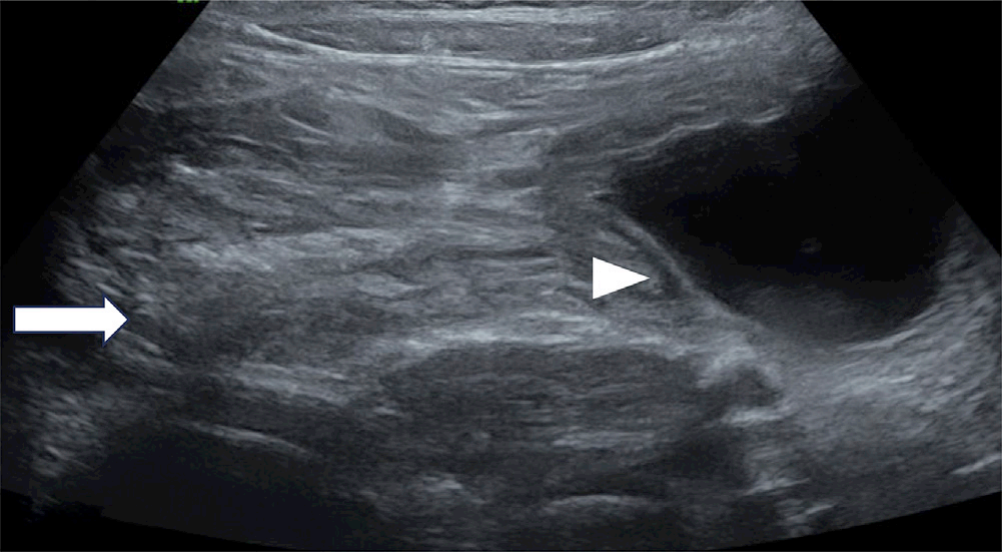
Longitudinal ultrasound image showing thickened bowel wall consistent with intussusception, demonstrating the characteristic “*pseudo-kidney*” appearance with alternating hypoechoic and hyperechoic layers (white arrow). Adjacent to this, a well-defined anechoic cyst with smooth walls is visible (white arrowhead), suggestive of a cecal duplication cyst.

**Fig. 3:**
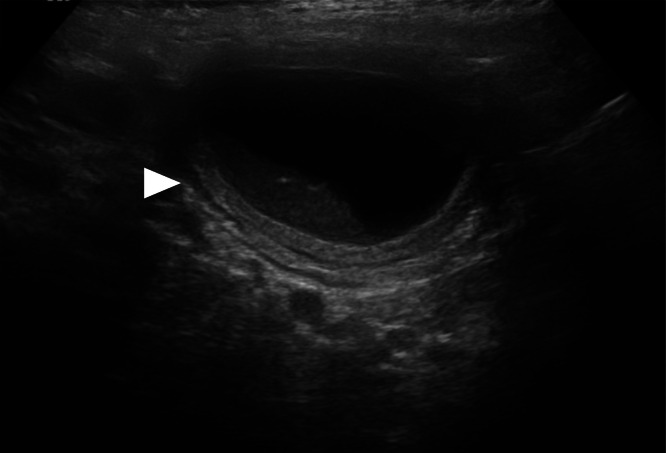
Longitudinal ultrasound image showing an oval, anechoic cystic lesion with well-defined borders and a hyperechoic “*double-wall”* appearance (white arrowhead). Internal fine echogenic debris is present in the dependent portion, and no vascularity is seen within the lesion on Doppler examination. The cyst is situated medial to the cecal wall.

**Fig. 4:**
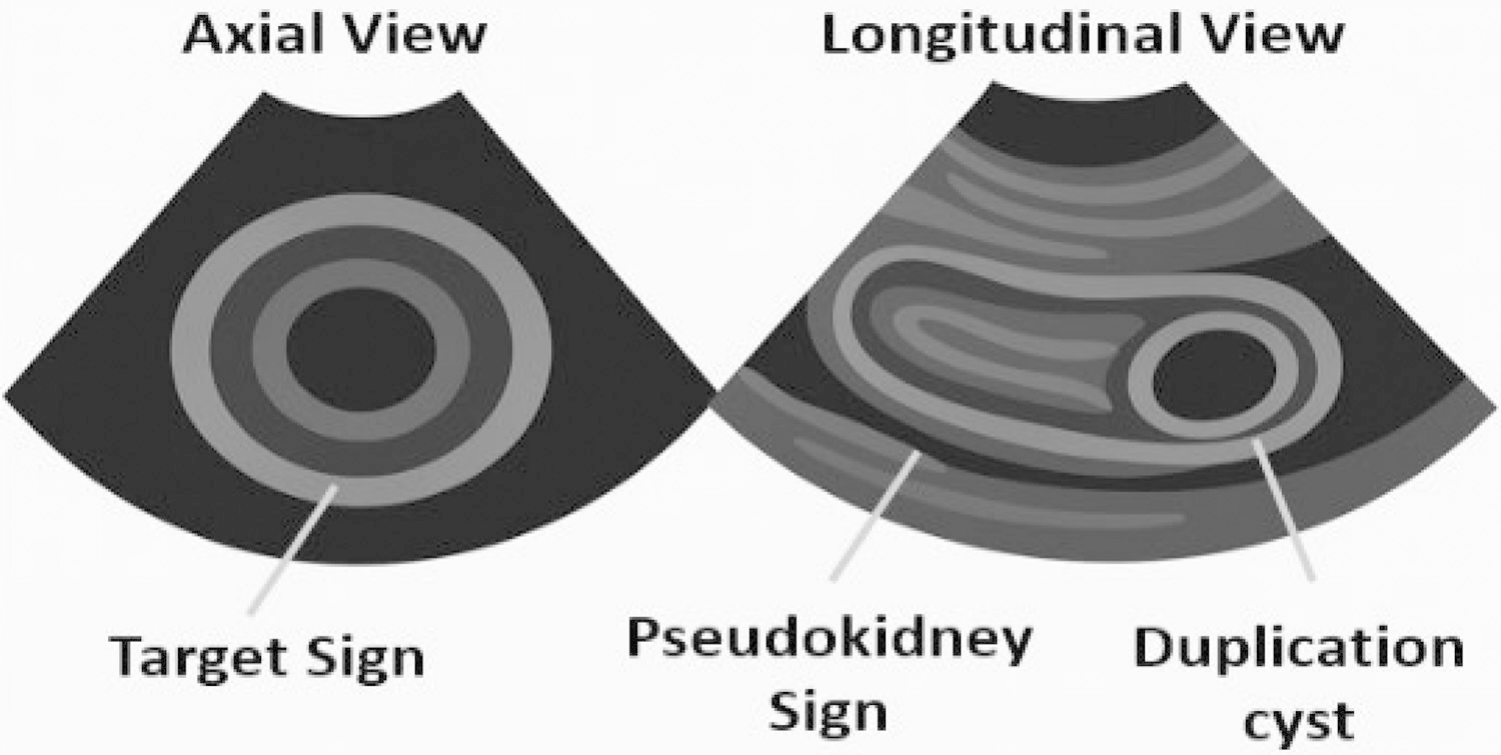
Schematic representation of axial and longitudinal ultrasound views of the intussusception. The transverse view demonstrates the characteristic “target sign,” while the longitudinal view shows the "pseudo-kidney" configuration. The adjacent cecal duplication cyst is also illustrated for correlation.

A 4-year-old girl presented to the emergency department with frequent episodes of intermittent abdominal pain over 4 months. The pain episodes did not involve gastrointestinal symptoms, such as vomiting, rectal bleeding, or signs of bowel obstruction. On examination, the abdomen was rounded, soft, and generally nontender, although discomfort was localized to the right iliac fossa. Bowel sounds were present and normal. Percussion revealed increased tympanism over the right lower quadrant. Routine laboratory tests returned results within normal limits. However, the patient's mother reported a brief course of antibiotic administration without medical supervision. The child appeared in good general health. Due to the persistence and localization of the abdominal pain, acute appendicitis was initially suspected. Clinicians performed an abdominal ultrasound without prior radiographic imaging. Sonographic examination revealed concentric alternating hypoechoic and hyperechoic rings in the transverse view, forming the classic “target” or “doughnut” sign, consistent with bowel wall invagination. This appearance reflects the presence of ileocolic intussusception, with the terminal ileum serving as the intussusceptum and the cecum as the intussuscipiens. In the longitudinal view, the bowel loop showed a reniform configuration, forming the so-called “pseudo-kidney” sign, with preserved layering of the wall. Adjacent to the intussusception, a well-defined anechoic cystic lesion was identified along the medial aspect of the cecum. The cyst measured 27 mm transversely and 36 mm craniocaudally, demonstrating fine internal echoes and dependent sediment, with no detectable vascularity on Doppler imaging. Its location and appearance were consistent with a cecal duplication cyst, and its position at the leading edge of the intussusceptum suggests it served as the anatomical lead point triggering the intussusception (Fig. 1, Fig. 2, Fig. 3, Fig. 4).

**Fig. 5:**
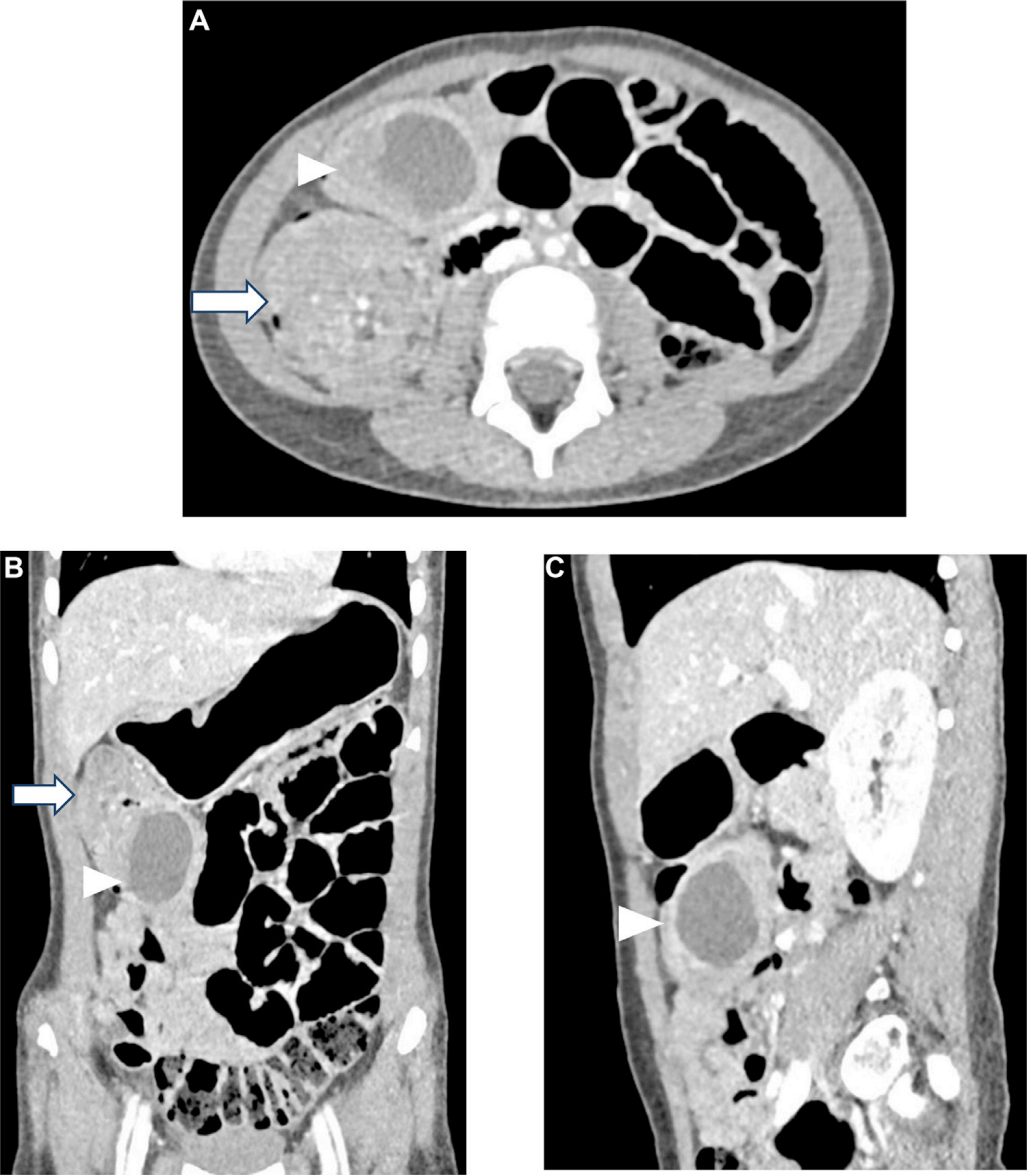
(A, B and C) Contrast-enhanced CT images of the abdomen in axial (A), coronal (B), and sagittal (C) planes demonstrate ileocolic intussusception characterized by a segment of bowel telescoping into the adjacent distal bowel, seen as an irregular, thickened bowel wall with layered enhancement (white arrows). Adjacent to this, a well-defined, low-attenuation cystic lesion with a thin, smooth wall is visualized (white arrowheads). This cystic structure corresponds to the cecal duplication cyst, lying close to the ileocecal region and distinct from the intussuscepted bowel segment.

To better characterize the lesion and support the diagnostic assessment, an abdominal CT scan was performed. The CT images demonstrated an ileo-colic intussusception, characterized by the classic “bull's-eye” or concentric ring appearance on both transverse and coronal views. A cystic mass containing fluid was visualized within the right iliac fossa and appeared closely associated with intussusception. The cyst measured approximately 22 mm in width, 27 mm in anteroposterior dimension, and 38 mm in craniocaudal length (Fig. 5).

The patient was taken to the operating room for exploratory surgery via a McBurney incision. Intraoperatively, an ileo-colic intussusception was identified along with a large cystic lesion with thickened walls. The cyst appeared to arise from the cecal region and did not visibly communicate with the bowel lumen. The intussusception was carefully reduced, and en bloc resection of the involved bowel segment was performed. This included the distal 3 cm of the ileum, cecum, and appendix, followed by primary end-to-end ileocolic anastomosis. The abdominal wall was closed in layers.

The patient was admitted to the pediatric surgery ward for postoperative monitoring. The postoperative course was uneventful, with return of bowel function on postoperative day 2 and resumption of oral feeding shortly thereafter. The patient was discharged on postoperative day 4 in good clinical condition. A follow-up visit 2 weeks later confirmed full recovery, with no signs of complications or recurrence.

**Fig. 6:**
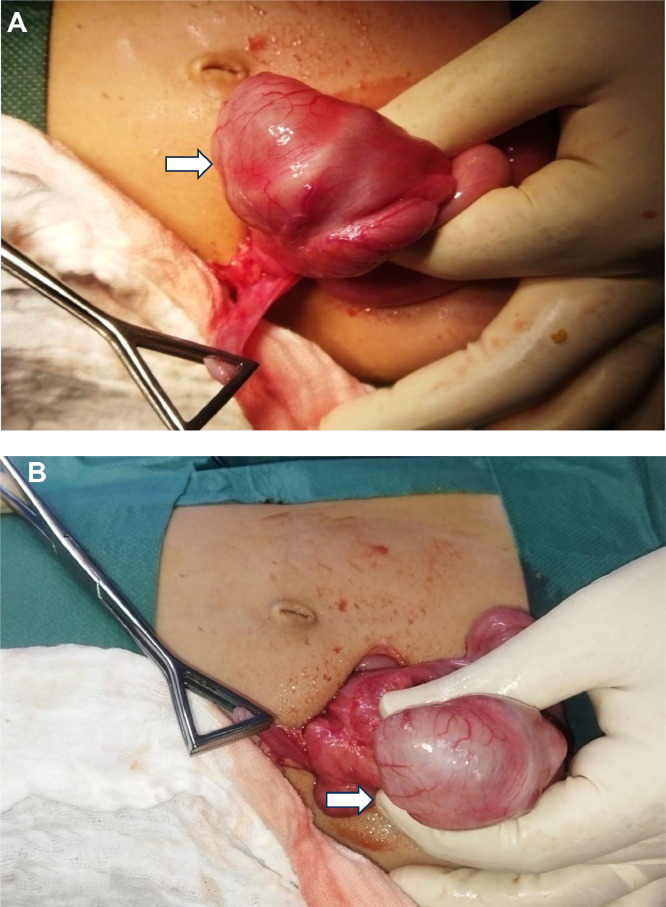
(A and B) Intraoperative images demonstrating a thick-walled cystic structure originating from the cecal wall. The lesion contained clear fluid and showed no communication with the bowel lumen. It was firmly adherent to the serosal surface of the cecum without evidence of invasion. The resected bowel segment reveals the cecal duplication cyst in continuity with the cecum, characterized by a smooth, well-defined cyst wall distinct from the adjacent bowel lumen, confirming its noncommunicating congenital nature consistent with a cystic duplication anomaly.

Macroscopic analysis revealed a cystic formation containing fluid within the cecum that was unconnected to the cecal lumen. Microscopic evaluation showed colonic mucosa lined by a regular mucosecretory epithelium, supported by a submucosal layer abundant in lymphoid follicles that exhibit distinct germinal centers (Fig. 6).

## Discussion

Enteric duplication is a rare congenital anomaly of the gastrointestinal tract that may occur anywhere along the alimentary canal from the oral cavity to the rectum. As described by Ladd and Gross, these duplications are typically closely associated with a segment of the native bowel, sharing its blood supply and featuring a smooth muscle layer and an epithelial lining similar to that of the adjacent digestive tract [7,8]. The ileum is the most commonly affected site (33%), followed by the esophagus (20%), colon (13%), jejunum (10%), stomach (7%), and duodenum (5%) [9,10]. Cecal duplication is particularly uncommon, with only a limited number of cases reported [11,12]. According to a review by Oudshoorn of 362 cases, only 16 involved the cecum [13]. The clinical presentation varies and may include abdominal pain, palpable mass, intussusception, gastrointestinal bleeding, or bowel obstruction, mimicking other acute conditions such as appendicitis. Anatomically, duplications are categorized as cystic or tubular, with cystic forms being more frequent [14]. Cystic duplications are typically noncommunicating and expand gradually owing to mucus accumulation, potentially leading to rupture, particularly when ulcerated ectopic gastric mucosa is present. They may also exert pressure on adjacent organs or vessels, causing inflammation and adhesion. Tubular forms are less common and often maintain communication with the GI tract; these are usually asymptomatic unless complications arise [15]. The exact embryological mechanism remains uncertain; however, several theories have been proposed, including vascular disturbances, incomplete fusion of embryonic folds, and persistence of embryonic diverticula during development [16]. Although generally benign, malignant transformation has been reported in rare cases, including adenocarcinomas originating within the duplication cyst wall [17]. Ultrasonography remains the first-line imaging modality, primarily because it is noninvasive and offers high diagnostic accuracy. The “double-wall” sign is highly suggestive of enteric duplication, while CT and MRI are used for further characterization when necessary [18,19]. In the present case, ultrasound detected a cystic mass with features consistent with intussusception, which was later confirmed using CT imaging. Management typically involves surgical resection and anastomoses when required. This approach offers favorable outcomes and minimizes the risk of recurrence or complications [20]. In cases involving intussusception, laparoscopic-assisted excision is a viable option; however, open surgery may be warranted depending on the intraoperative findings and the anatomical relationship between the cyst and adjacent bowel structures [21]. In our case, open surgical exploration enabled both diagnosis and resection of the affected ileal wall, followed by enterorrhaphy to preserve the ileocecal valve—a technique considered safe and effective according to previous studies [22]. Timely diagnosis and complete excision are crucial for preventing complications such as obstruction, perforation, or malignancy, and the overall prognosis is excellent when treatment is not delayed [22].

## Conclusion

Cecal duplication cysts are rare and often pose a diagnostic challenge because of their nonspecific symptoms and resemblance to more common abdominal pathologies, such as appendicitis. Early recognition through imaging, particularly ultrasonography, is essential to guide timely intervention. Surgical excision remains the definitive treatment, offering excellent outcomes when promptly performed. Clinicians should consider enteric duplication in the differential diagnosis of persistent or atypical abdominal pain in pediatric patients, especially when findings such as intussusception are present.

## Patient consent

I, the author of the article *“*Intussusception caused by cecal duplication cyst mimicking appendicitis: a case report,*”* declare that the patient provided informed consent for the publication of the case details, images, and related information at the time they were obtained.

## References

[1] Liaqat N., Latif T., Khan F.A., Iqbal A., Nayyar S.I., Dar S.H. (2014). Enteric duplication in children: a case series. J Paediatr Surg.

[2] Sookram J., Naidoo N., Cheddie S. (2016). Perforated cecal duplication cyst presenting as an appendicular abscess. Afr J Surg.

[3] Balakrishnan K., Fonacier F., Sood S., Bamji N., Bostwick H., Stringel G. (2017). Foregut duplication cysts in children. JSLS.

[4] Ko S.Y., Ko S.H., Ha S., Kim M.S., Shin H.M., Baeg M.K. (2013). A case of a duodenal duplication cyst presenting as melena. World J Gastroenterol.

[5] Verma S., Bawa M., Rao K.L., Sodhi K.S. (2013). Cecal duplication cyst mimicking intussusception. BMJ Case Rep.

[6] Anand S., Aleem A. (2022).

[7] Ladd W.E. (1937). Duplication of the alimentary tract. South Med J.

[8] Gross R.E. (1953).

[9] Cinta S.N., Roberto L.S., Elena C.P. (2018). Enteric duplication cysts in children: varied presentations and imaging findings. Insights Imaging.

[10] Roy L., Douglas G.A. (2014). Duplication cysts: diagnosis, management and the role of endoscopic ultrasound. Endosc Ultrasound.

[11] Mehl S.C., Anbarasu C., Sun R., Naik-Mathuria B. (2020). Cecal duplication cyst: a rare cause of pediatric bowel obstruction. AmSurg.

[12] Pati A., Mohanty H.K., Subudhi P.C. (2010). Duplication cyst of the cecum: a case report. Indian J Surg.

[13] Oudshoorn J.H., Heij H.A. (1996). Intestinal obstruction caused by duplication of the cecum. Eur J Pediatr.

[14] Saxena R., Pathak M., Sinha A. (2020). Cecal duplication cyst: a rare disease with variable presentation and its management in the era of laparoscopy. J Minim Access Surg.

[15] Keum S., Hwang M.W., Na J., Yu S., Kang D.B., Oh Y.K. (2009). Intestinal obstruction caused by a duplication cyst of the cecum in a neonate. Korean J Pediatr.

[16] Stringer M.D., Spitz L., Abol R., Kely E., Drake D.P., Agrawal M. (1955). Management of alimentary tract duplication in children. Br J Surg.

[17] Pati A., Mohanty H.M., Subudhi P.C., Dash R., Mohanty P.K., Mahapatra R.K. (2010). Duplication cyst of the cecum: a case report. Indian J Surg.

[18] Radhakrishna V., Rijhwani A., Jadhav B. (2018). Cecal duplication: a mimicker of intussusception: a case report and review. Ann Med Surg (Lond).

[19] Verma S., Bawa M., Rao K.L., Sodhi K.S. (2013). Cecal duplication cyst mimicking intussusception. BMJ Case Rep.

[20] Khalid A., Tahir A., Ashraf M., Masood A., Waqar M., Saeed F. (2022). Cecal duplication cyst mimicking intussusception in a female: a case report. Int J Surg Case Rep.

[21] Al-Shaibi M.A., Raniga S.B., Asghar A.N., Al Tubi I.S. (2019). Cecal duplication cyst leading to intussusception in an adult. BMJ Case Rep.

[22] Catalano P., Di Pace M.R., Caruso A.M., De Grazia E, Cimador M. (2014). Ileocecal duplication cysts: is the loss of the valve always necessary?. J Pediatr Surg.

